# Childhood pesticide poisoning in Zhejiang, China: a retrospective analysis from 2006 to 2015

**DOI:** 10.1186/s12889-017-4505-3

**Published:** 2017-06-28

**Authors:** Aziguli Yimaer, Guangdi Chen, Meibian Zhang, Lifang Zhou, Xinglin Fang, Wei Jiang

**Affiliations:** 1 Department of Public Health, Zhejiang University School of Medicine, Hangzhou, 310058 China; 2 Department of Environmental and Occupational Health, Zhejiang Provincial Center for Disease Control and Prevention, Hangzhou, 310051 China

**Keywords:** Pesticide poisoning, Children, Retrospective analysis, Organophosphate

## Abstract

**Background:**

Pesticide poisoning in children has been a serious public health issue around the world, especially in the developing countries where agriculture is still one of the largest economic sectors. The purpose of this study was to analyze epidemiological characteristics of acute pesticide poisoning in children from Zhejiang province, China.

**Methods:**

The pesticide poisoning cases for children were retrieved from Occupational Disease Surveillance and Reporting System, Zhejiang Provincial Center for Disease Control and Prevention, China. The incident cases, deaths, and fatality rate of child pesticide poisoning from 2006 through 2015 were calculated.

**Results:**

During the study period, totally 2952 children were poisoned by pesticides, with 66 deaths, resulting in a fatality rate of 2.24%. Among them, there were 1607 male cases with 28 deaths, and 1345 female cases with 38 deaths. Most of the cases occurred in preschool children (1349) and adolescent age group (1269). Organophosphate and carbamate insecticides were the cause of most poisonings (1130), leading to 34 deaths. The highest fatality rate (3.13%) was due to poisoning by herbicides and fungicides, causing 14 deaths out of 448 cases. Poisoning occurred mostly in rural areas (78%). And most pesticide poisoning occurred in the summer (896) and fall (811), while fewest poisoning cases in the winter (483) but with the highest fatality rate (3.52%).

**Conclusions:**

This study shows that pesticide poisoning of children is a major health problem in Zhejiang, suggesting preventive strategies should be conducted to control childhood pesticide poisoning.

## Background

Pesticide is a collective term for a wide array of chemicals that refers to insecticides, herbicides, fungicides, rodenticides, etc. [1]. Different types of pesticides are applied to kill insects, rodents, fungi, weed and also used to kill vectors of diseases in public health [2]. Due to the widespread and inappropriate use of pesticide, pesticide poisoning stands as a serious health problem and remains a significant public health issue. According to WHO data, pesticide poisoning accounts for an estimated 250,000 deaths annually among three million cases worldwide [3]. Notably, a large proportion of studies on pesticide poisoning were carried out in adults [4–6]. However, pesticides are common poisoning reasons for children especially in the developing countries [7]. In Egypt from 2009 to 2013, pesticides were the most common agents resulting in moderate or severe toxicity from non-pharmaceutical exposures in children, as well as the most common reason for admission to hospital [8]. In South African pesticide poisoning accounted for 11% of all paediatric poisoning cases (2003–2008) [9]. Childhood pesticide poisoning accounted for 37% of all pesticide poisoning calls reported to the National Poison Information Centre of India from 1999 to 2012 according to the All India Institute of Medical Science [10].

Different to adult pesticide poisoning, childhood pesticide poisoning has some characteristics. Firstly, contaminated food and water, and pest control in the home, yard, and school are all potential sources of children’s exposure [11]. Children’s behaviors and abilities to interact with their physical environment change during the different stages of growth and development, which can place them at greater risk [12]. Secondly, children are more vulnerable than adults to pesticide exposure [12, 13]. Children have rapid growing and developing organ systems, and the long-lasting exposure to pesticides during childhood have been associated with an increased risk of malignancy in adulthood [14, 15], respiratory symptoms [16], delays or impairments in language development [17, 18] and other neurodevelopmental outcomes [19–21]. The health effects of pesticide poisoning for children are an ongoing focus of concern and inquiry. Thus, it is vitally important to pay more attention to child pesticide poisoning.

 In China, the pesticide poisoning is one of the major public health issues [6, 22–25]. However, the epidemiological information about pesticide poisoning among children in China is limited. The Occupational Disease Surveillance and Reporting System (ODSRS) has been launched since 2006, which archives registration cases of occupational diseases and pesticide poisoning. Our previous study reported pesticide poisoning and death in adults with age over 20 years previously using the data provided by this system [26]. In this study, we described the overall epidemiological characteristics of acute childhood pesticide poisoning using the data from pesticide poisoning cases registered with the ODSRS in Zhejiang province, China. This work was a necessary complement to previous report and also provided important messages about pesticide poisoning of children, which should not be ignored. To the best of our knowledge, this is the first retrospective analysis of pesticide poisoning among children in Zhejiang, China.

## Methods

### Ethics

All the patient information data required in our study was encrypted in the official pesticide poisoning statistics and ODSRS by the Zhejiang Provincial Center for Disease Control and Prevention (CDC) China. The patients’ privacy was protected. Our study complied with the Declaration of Helsinki and was exempted from institutional ethical review by the Research Ethics Board of Zhejiang Provincial CDC.

### Data source

Acute childhood pesticide poisoning data from 2006 to 2015 were obtained from ODSRS at the Zhejiang Provincial CDC. The data collection technique used in our research has been described previously [26]. Briefly, the reporting system was established in 2006 and already covered 395 hospitals and community healthcare centers in cities, and clinics in rural areas. Consequently, the patients covered in this study represent both urban and rural populations. The child pesticide poisoning and death certification were required by Zhejiang CDC and reported by physicians from reporting system healthcare institutions. Data obtained included basic demographics regarding each patient including age (a child is considered to be 18 years old or less), dates of hospitalization, address of medical institution and other factors.

### Data analysis

Age of the children was divided into four groups: an infant group (less than 1 year), a pre-school age group (1–6 years), a school age group (7–12 years) and an adolescent group (13–18 years) [27]. The official death statistics and the database of ODSRS employ the International Classification of Disease, Ninth version, Clinical Modification (ICD-9-CM) coding system in China [26]. The data were tabulated in Microsoft Office Excel and statistically analyzed using SPSS statistics software (Version 16.0, SPSS Inc., Chicago, IL). Figures were prepared with GraphPad Prism software (Version 5.01, GraphPad software Inc., La Jolla, CA).

## Results

### Pre-school and adolescent groups accounted for most of the poisoning cases

Table 1 shows the pesticide poisoning case number, deaths and fatality rate yearly from 2006 to 2015. The annual cases and deaths remained steady so we combined all cases as study population to present the overall epidemiological characteristics of pesticide poisoning among children in Zhejiang province in ten years. Totally, 2952 child pesticide poisoning cases were identified, with 66 death cases, which led to a fatality rate of 2.24% (Table 2). There were 1607 male poisoning cases and 1345 female cases, which contributed 54% and 46% to the total child pesticide poisoning, respectively. The overall proportion of male to female cases of poisoning during the study period was 1.19 to 1. As shown in Table 2, more cases were found in rural areas (2296 for 77.8%) compared to urban areas (656 for 22.2%). Rural-urban ratios of incidents and fatalities were 3.5 to 1 and 1.06 to 1, respectively. There was slight difference in the number of cases between flatlands (1531 for 51.9%) and hills (1421 for 48.1%). But the fatality rate in the flatlands (2.94%) was nearly twice that in the hills (1.48%). The highest fatality rate was observed in infants (4.17%), followed by adolescents (3.78%).

**Table 1:** Annual reported pesticide poisoning among children in Zhejiang province, China

Year	Incident Number			Death Number			Fatality rate (%)		
	Male	Female	Total	Male	Female	Total	Male	Female	Total
2006	86	57	143	2	3	5	2.33	5.26	3.50
2007	152	157	309	4	5	9	2.63	3.18	2.91
2008	178	153	331	3	7	10	1.69	4.58	3.02
2009	182	147	329	1	6	7	0.55	4.08	2.13
2010	180	127	307	4	4	8	2.22	3.15	2.61
2011	181	154	335	3	2	5	1.66	1.30	1.49
2012	199	148	347	4	3	7	2.01	2.03	2.02
2013	179	171	350	3	5	8	1.68	2.92	2.29
2014	138	119	257	2	1	3	1.45	0.84	1.17
2016	132	112	244	2	2	4	1.52	1.79	1.64

**Table 2:** Demographic characteristics of pesticide poisoning among children in Zhejiang province, China

	Incident Number	Death Number	Fatality rate (%)
Total	2952	66	2.24
Sex			
Male	1607	28	1.74
Female	1345	38	2.83
Age group			
Infant	48	2	4.17
Pre-school	1349	12	0.89
School	286	4	1.40
Adolescent	1269	48	3.78
Area			
Urban	656	14	2.13
Rural	2296	52	2.26
Geography			
Flatlands	1531	45	2.94
Hills	1421	21	1.48
Season			
Spring	762	9	1.18
Summer	896	21	2.34
Fall	811	19	2.34
Winter	483	17	3.52

**Fig. 1:**
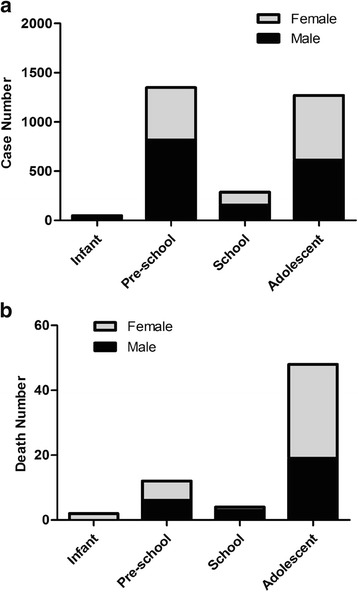
Childhood pesticide poisoning cases and deaths in Zhejiang from 2006 to 2015 among children by different age group. **a** Number of pesticide poisoning cases in different age groups; (**b**) Number of pesticide poisoning deaths in different age groups

**Fig. 2:**
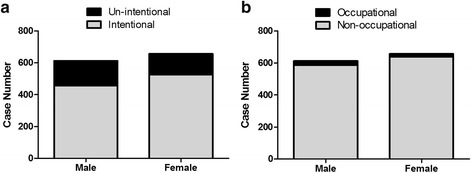
Childhood pesticide poisoning cases in Zhejiang from 2006 to 2015 in adolescents. **a** Intentional/un-intentional distribution in both genders; (**b**) Occupational/non-occupational distribution in both genders

In our study, the most affected age group was pre-school (1349 for 46%) followed by adolescents (1269 for 43%),the school age group (286 for 9.7%) and infants (48 for 1.6%). Most of the death cases occurred in adolescents (48 for 72.7%). As showed in Fig. 1, the distribution of pesticide poisoning by age group in both male and female groups. Similar distribution pattern was found in both boys and girls. For both gender groups, most pesticide poisonings occurred in pre-school children and adolescents with most of the deaths in the adolescent group. More boys were poisoned in all the age groups except for the adolescent group (Fig. 1a). More female deaths were found in the adolescent and infant groups (Fig. 1b). For adolescent group, most of the cases were intentional and non-occupational in both male and female (Fig. 2).

### Child pesticide poisoning was common during the farming season

**Fig. 3:**
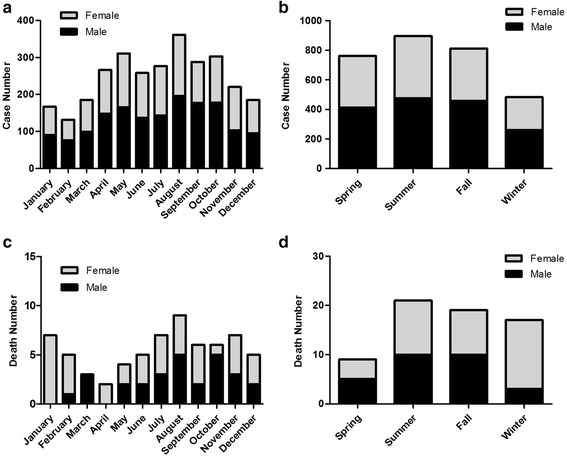
Childhood pesticide poisoning cases and deaths in Zhejiang from 2006 to 2015 by month and season. **a** Monthly pesticide poisoning cases; (**b**) Seasonal pesticide poisoning cases; (**c**) Monthly pesticide poisoning deaths; (**d**) Seasonal pesticide poisoning deaths

Seasonal variation was found in the number of child pesticide poisoning cases. Fig. 3 shows the distribution of the cases and deaths by month and season in male and female groups. According to the distribution of poisoning cases in different months, child pesticide poisoning was most common in August with 361 cases and least in February with 131 cases (Fig. 3a). From the perspective of the seasons, more cases were reported in farming season: summer (896 for 30.4%) and fall (811 for 27.5%) (Fig. 3b). Same distribution pattern of monthly and seasonal poisoning cases was found in both male and female groups. Cases involving boys outnumbered those involving girls all seasons (Fig. 3b) and the same pattern was observed except for November when analyzed by month (Fig. 3a).

The monthly child pesticide poisoning deaths ranged from 2 to 9 cases, in which the number of deaths was highest in August and lowest in April (Fig. 3c). Most deaths in boys occurred in August and October. The highest number of deaths in girls was observed in January. On the other hand, most deaths occurred during summer and fall in boys, while most deaths occurred during winter in girls (Fig. 3d). Taken together, there were 40 deaths during summer and fall, which accounted for 60.6% of all death cases.

### Organophosphate and carbamate insecticides was the most common type in child pesticide poisoning

A break-down of poisoning incidents versus the type of pesticide is given in (Table 3). Poisoning by organophosphate and carbamate insecticides was most common: there were 1130 cases, which accounted for 38.3% of all cases with 3.01% fatality rate. Overall, insecticides were the main agent responsible for child pesticide poisoning (1846 for 62.5%). There were 448 cases caused by herbicide and fungicide poisoning with the highest fatality rate (3.13%), in which all death cases were due to paraquat poisoning. The pesticides involved in most deaths were dichlorvos (15 for 22.7%), paraquat (14 for 21.2%) and Methamidophos (8 for 12.1%).

**Table 3:** The pesticide types involved in childhood pesticide poisoning in Zhejiang province, China

	Incident Number	Death Number	Fatality rate (%)
Total	2952	66	2.24
Organophosphate and carbamate insecticides	1130	34	3.01
Dichlorvos	456	15	3.29
Methamidophos	181	8	4.42
Parathion	18	0	0.00
Omethoate	108	4	3.70
Trichlorfon	32	1	3.13
Isocarbophos	13	0	0.00
Other organophosphates	267	6	2.25
Carbofuran	18	0	0.00
Other carbamates	24	0	0.00
Methomyl	13	0	0.00
Halogenated insecticides	132	1	0.76
Fluoroacetamide	24	0	0.00
Deltamethrin	108	1	0.93
Other insecticides	584	8	1.37
Pyrethroid	257	1	0.39
Organochlorine	20	1	5.00
Other insecticides	222	4	1.80
Chlordimeform	19	0	0.00
Dimehypo	66	2	3.03
Herbicides and fungicides	448	14	3.13
Paraquat	212	14	6.60
Other herbicides	188	0	0.00
Fungicides	48	0	0.00
Rodenticides	524	6	1.15
Tetramine	91	3	3.30
Other rodenticides	228	2	0.88
Anticoagulant rodenticides	205	1	0.49
Others	134	3	2.24
Multipurpose formulation	32	0	0.00
Biochemical pesticide	19	0	0.00
Others unspecified	83	3	3.61

## Discussion

We analyzed the epidemiological characteristics of child pesticide poisoning in this study and our work revealed that pesticide poisoning occurred mostly in rural areas which was consistent with reports in China and other countries [28–30]. This was attributed to common pesticide usage related to agricultural activities in these areas. Pesticides are easily available to rural families.

Organophosphate and carbamate insecticides were the most common pesticides causing child pesticide poisoning in our study, which may be due to their common usage in agriculture and hence their easy availability. Most deaths were from poisoning by dichlorvos, which is one of the most commonly used organophosphates in China [24]. During the study period, 14 deaths were caused by paraquat, which is a highly efficient herbicide with intense toxicity for humans. Treating paraquat poisoning is very difficult because no specified antidotes exist [31, 32]. A ban on the production of liquid paraquat took effect in July 2014 and the sale for agricultural use was banned in July 2016 in China. The death rate from herbicides is expected to drop in the next a few years due to this policy.

Our study found that childhood pesticide poisoning was common in the farming season in Zhejiang. In our previous study, we also found more pesticide poisoning and death cases in adult during the farming season in Zhejiang province [26]. Studies in other countries showed the incidence of pesticide poisoning correlated with pesticide availability due to the season-specific agricultural activities [9, 29, 33].

Out of all age the groups, pesticide poisoning of infants yielded the highest fatality rate. Infant pesticide poisoning is rare because they are taken good care of by adults with intensive attention and they have limited ability to get access to pesticides by themselves. Most of the cases are due to consumption of contaminated food [34]. The highest fatality rate in the infant group might be due to their particular vulnerabilities.

The highest frequency of pesticide poisoning was found in the pre-school age group which was consistent with other studies among children [8–10, 35, 36]. This could be due to their inherent inquisitiveness and high “hand to mouth” activity out of curiosity and their exploratory nature [37]. It is an incontrovertible fact that children are curious about their surroundings and are often unaware of the impending danger. Children at this age may be able to gain access to pesticides, but they have no developed cognitive hazard awareness. The chance of accidental poisoning increased when the pesticides were not safely stored [10, 33, 38, 39]. Pesticide poisoning of children is mostly preventable if effective preventive strategies are employed. It is important to provide children with a safe environment. This requires better management of pesticides, including labeling of pesticide containers with poison warning stickers, and immediately placing pesticides into safe storage after use. In most developed countries child-resistant packaging has been used for packaging of medications, household chemicals and pesticides, which has proven to be one of the most effective preventive measures against unintentional poisoning of young children [11]. In China, child-proof caps are not widely used in the packaging of pesticides, which makes pesticides more accessible to kids. Therefore, child-resistant packaging and closures are strongly recommended for pesticide manufacturing and storing in China.

Case numbers dropped significantly for children of school age when they became more aware of pesticide risks and spent less time in unsafe home environments. However, poisoning case numbers rebounded for the adolescent group with an increase also in the fatality rate. Other studies have also found that case number and fatality rates decreased with age, but then increased to a second peak during adolescence [27, 38]. This was likely related to intentional poisoning aimed at self-harm. Such exposures are an important social problem among adolescents in some Asian countries, including China [40]. In our study, most of the cases in adolescents were intentional. Thus, suicide by pesticide in adolescents has been a serious public health problem in Zhejiang. More attention should be paid to the mental health of adolescent group.

More boys were poisoned than girls in numbers in our study, which was similar to previous studies [9, 33, 35], probably as a result of the higher activity of boys. We found female cases were more common in the adolescent group resulting in a conspicuously high number of deaths. These findings were consistent with other studies that found significant associations between adolescent girls and intentional poisoning [41–43]. According to those studies, the higher rate of suicide in adolescent females was correlated with depressive symptoms and romantic disappointment. These conditions were more prominent in girls than in boys. Overall, suicide remains a leading cause of death in adolescent and the ingestion of pesticide is among the most common methods of suicide globally.

Our study shows that it is possible to monitor cases of pesticide poisoning using ODSRS. This database is valuable from public health perspective and for long term monitoring of the effects of pesticides. Still, there are some limitations of this register-based study. The observations in this report are empirical, that is they are based on experience and not on theory or logic. There is no other way to gather this sort of information. The empirical nature of these results does not reveal the causes for the observations. This is an inherent limitation of empirical approach. The nature of our study was descriptive epidemiology. The absolute number could provide some useful information from public health perspectives. Another major limitation of our study is the ODSRS is hospital based and a number of rural clinics are still not included as surveillance sites. Pesticide poisoning was much more common in rural areas. This led to an unknown total population number. In addition, misdiagnosis was often unavoidable due to the lack of useful descriptions about the symptoms of sick children. There might be some out-of-hospital deaths caused by pesticide poisoning which also resulted in under-reporting. And people in severe cases will be transferred to provincial hospitals from rural clinics for better therapy. They are often registered as “survival” instead of being followed up. In addition, some physicians in rural areas may fail to report cases to the system. We will say that the establishment of the ODSRS has improved the reporting rate, but under-reporting still exists. We didn’t register the types of factors leading to poisoning currently. To improve the system and get more useful information, we will suggest add types of poisoning to the data base. For example, some common reasons for unintentional poisoning: inadequate storage places or inappropriate ways of storage for pesticides etc. The detailed information would help us to make strategies to prevent pesticide poisoning of children. We only included child pesticide poisoning in Zhejiang province in this study, which may not represent pesticide poisoning patterns in other provinces in China. Future studies about the reporting rate by the ODSRS in other areas are warranted to better estimate the child pesticide poisoning in China.

## Conclusions

The present study provided description of childhood pesticide poisoning in Zhejiang province, China from 2006 to 2015. Our results showed that child pesticide poisoning is a serious health problem in Zhejiang and most of the cases occurred in preschool children, which indicated a need for child-resistant packaging and closures. Restrictive measures should be taken to control the use and storage of pesticides, especially in rural areas. Attentions should be paid to the mental health of adolescents to reduce their self-harm behaviors and more effort is needed to increase public awareness of safety for the use of pesticides. A ban on usage is suggested for those pesticides with high toxicity toward humans.

## Abbreviations

CDC: Center for Disease Control and Prevention
ODSRS: The Occupational Disease Surveillance and Reporting System
